# Modelling the dynamics of growth, development and lipid storage in the marine copepod *Calanus finmarchicus*

**DOI:** 10.1007/s00227-016-3030-8

**Published:** 2016-11-22

**Authors:** Tjalling Jager, Iurgi Salaberria, Dag Altin, Trond Nordtug, Bjørn Henrik Hansen

**Affiliations:** 1 DEBtox Research, De Bilt, The Netherlands; 2 Department of Biology, Norwegian University of Science and Technology, Trondheim, Norway; 3 BioTrix, Trondheim, Norway; 4 SINTEF Materials and Chemistry, Marine Environmental Technology, Trondheim, Norway

## Abstract

Mechanistic models are essential tools for interpreting and predicting the consequences of a changing environment and stressors such as pollution on the life histories of marine organisms. Here, we apply the simple and generic energy-budget model DEBkiss to the life history of the marine copepod *Calanus finmarchicus*. Model modifications were needed to accommodate the copepod life cycle, which deviates in several respects from most other animals (e.g., a sudden stop of growth after the final moult). We identified an acceleration of growth in the early copepodite stages, which could be linked to an increase in the specific feeding rate of the animals. Lipid storage, an essential element of *C. finmarchicus* biology, was successfully captured with the reproduction buffer of the DEBkiss model. The resulting model was fitted to a detailed data set from the literature and was able to explain growth, development and lipid storage from egg to adult, at different temperatures and food availabilities, within a single consistent framework. The parameterised model could subsequently be used to elucidate the energetic constraints on gonad maturation and reproduction. Interestingly, the overhead costs for egg production seem to be substantially higher than the default value applied in DEB-based studies. The current model provides a solid basis for applications in stress ecology, although our model analysis also identified several knowledge gaps. Specifically, further research is needed to cover the dynamics of diapause and gonad maturation, to explain the dependence of maximum body size on food and temperature, and to verify the predicted high costs for maturity maintenance.

**Electronic supplementary material:**

The online version of this article (doi:10.1007/s00227-016-3030-8) contains supplementary material, which is available to authorised users.

## Introduction

Calanoid copepods form an important part of the marine zooplankton. To interpret and predict the impact of stressors, such as pollution and climate change, on their life histories, models at the individual level are essential tools. Life-history traits, and the effects of stressors on these traits, will depend on the (time-varying) environmental conditions such as food availability and temperature. As we cannot hope to test all relevant scenarios in the laboratory, we need models with a mechanistic basis to extrapolate to field conditions. A natural way to build such models is to focus on the energy budget, because all organisms need to obey the conservation laws for mass and energy. Dynamic energy budget (DEB) theory (Kooijman 2010; Nisbet et al. 2000) offers a coherent set of simple rules, describing how organisms take up food from the environment, and how the assimilated resources are allocated to fuel all energy-requiring processes such as growth, maintenance and reproduction, over the entire life cycle (from egg to adult). Stressors, such as toxicants and ocean acidification, can affect these bioenergetic processes and thereby lead to changes in the life history (Jager and Zimmer 2012; Jager et al. 2016).

A major advantage of DEB theory is that it is not species specific; the theory covers all life forms, and the same basic model structure can be used for most animal species (Lika et al. 2011). Copepods, however, have several features in their life cycle which require further consideration in energy-budget models. Firstly, they develop through six naupliar stages, which deviate in morphology from the later six copepodite stages, and the first naupliar stages do not feed. Secondly, copepods exhibit true determinate growth and stop growing rather abruptly after their final moult to adulthood. And thirdly, many copepods (especially species from higher latitudes) build up a considerable lipid storage during the last few copepodite stages to cope with fluctuating environmental conditions (such as low food availability) and to fuel the reproduction process (Lee et al. 2006).

Despite the power and generality of DEB theory, many eco(toxico)logical applications are better served by simpler representations of bioenergetics (Nisbet et al. 2010; Jager et al. 2013). In a previous study (Jager et al. 2015), we demonstrated how the simplified energy-budget framework DEBkiss (Jager et al. 2013; Jager 2015) could be used to capture some aspects of the life history of *Calanus sinicus*. The first two (non-feeding) naupliar stages were treated as a continuation of the embryo stage; their energetic requirements are covered by the yolk provided by the mother in the egg. The feeding naupliar stages behaved the same as the copepodite stages, after accounting for their difference in shape. The determinate growth was included as a switch in energy allocation after the final moult: investment into growth is stopped and the surplus energy is used for reproduction. Lipid storage was, however, not treated in the previous work, mainly due to a lack of suitable data. This process is nevertheless of great importance in many copepod species. If environmental or anthropogenic stress implies that less energy can be allocated to lipid storage, this could have severe consequences for survival during winter, for the timing of reproduction, and for the total reproductive output. These factors may in turn affect recruitment of fish species such as cod, whose larvae critically depend on copepods as food source (Beaugrand et al. 2003). Furthermore, differences in lipid content are associated with differences in body burdens of hydrophobic chemicals, and sensitivity to their toxic effects (Hansen et al. 2016).

In this study, we examine the bioenergetics of *Calanus finmarchicus* over its full life cycle, with special emphasis on lipid storage. *C. finmarchicus* is a dominant zooplankton species in the Northern Atlantic Ocean and expanding up into the Arctic. This species is also used for toxicity testing in relation to oil pollution in the marine environment (e.g., Hansen et al. 2013, 2016). Campbell et al. (2001) present an extensive data set on the life history of *C. finmarchicus* under laboratory conditions, including the effects of temperature and food limitation. We will use this data set to test the DEBkiss model previously developed for *C. sinicus*, and to expand the model to include the lipid storage. The primary aim of this study is to quantitatively understand the entire suite of life-history traits of *C. finmarchicus* within the constraints of the conservation laws. However, working in a DEB context, we need to constrain ourselves further by demanding consistency with the metabolic rules for other animals. All animals are, metabolically speaking, quite similar (Lika et al. 2011), and there is a lot to be gained from including *C. finmarchicus* within this big picture, rather than developing a radically different model for each species (or each group of species). For example, this larger framework allows us to meaningfully compare species across large taxonomical distances, as was demonstrated for the DEBkiss model by Jager and Ravagnan (2016). A coherent energy-budget formulation is clearly of scientific interest. However, once this model is established and tested, it can be used in future studies as a platform to interpret and predict the effects of stressors such as toxicants.

## Methods

### Data set and life-history observations

The data set of Campbell et al. (2001) contains measurements of nitrogen (N) and carbon (C) in *C. finmarchicus* over time (from egg to adult), at three temperatures, as well as two limiting food levels. This data set is particularly useful to examine the energetics of lipid storage as nitrogen can be considered a proxy for structural body mass, whereas carbon combines contributions from structure and lipid storage. From the publication of Campbell et al. (2001), we can also derive several general observations on the life history of *C. finmarchicus* that the model would need to capture, and preferably explain. These observations are summarised in Table 1 and will be referred to in the text below.

Additionally, we used data sets for respiration in C5 from Clarke and Bonnet (1939), ingestion and reproduction rates from Båmstedt et al. (1999) for adults, and reproduction rates from Rey et al. (1999). Data on filtration and ingestion rates for different stages were taken from Marshall and Orr (1956) and Meyer et al. (2002). Data were extracted from the graphs in the original publications using the freeware PlotReader (http://jornbr.home.xs4all.nl/plotreader).

We require a number of conversion factors to work with various body size measures ($d_{\rm C}, d_{\rm N}, d_{\rm V}, \delta _{\rm M}$) and to translate one flux in another (the yield coefficients $y_{\rm VA}, y_{{\rm BA}}$ and $y_{\rm AXc}$). The values for these conversions, and the egg weight ($W_{\rm B0}$), are provided in Table 3 (rationale for these choices given in supp. info.). The set of conversion factors is quite comparable to that established for *C. sinicus* earlier (Jager et al. 2015), facilitating comparison between these species.

**Table 1:** General observations on life-history patterns in *C. finmarchicus* (based on Campbell et al. 2001)

	Observed pattern
1	Structural growth stops after the final mould to C6
2	Nauplii grow slower than the copepodites (relative to the expected von Bertalanffy pattern)
3	At higher test temperatures, size at a given stage is smaller than at lower temperatures (at least for copepodites)
4	At limiting food levels, maximum structural size is smaller (both in terms of length and N content)
5	Lipid storage becomes evident in the third copepodite stage (C3), and the lipid fraction (indicated by the C/N ratio) increases over time
6	Initiation of lipid storage has no negative impact on structural growth
7	At low food levels, little or no lipid storage is built up
8	Lipid storage declines after the final moult (at least at higher temperatures)

### DEBkiss model for copepods

**Fig. 1:**
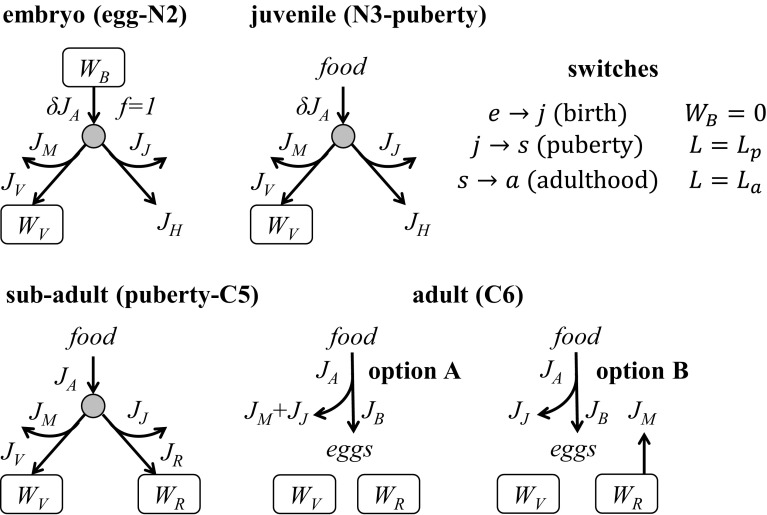
The energy flows in the modified DEBkiss model for each of the four metabolic life stages of *C. finmarchicus*. Fluxes ($J_i$ in mg/day) and states ($W_i$ in day) for each process are explained in Table 2. Switches between the life stages are based on the values of the state variables for egg buffer or body size. The allocation schemes for the adult stage are preliminary and used for the predictions in Fig. 7. *Filled circle* represents the $\kappa$-rule allocation, with a fraction $\kappa$ of the assimilation flux allocated towards the soma (structural growth and somatic maintenance). Note that $J_{\rm H}$ is completely dissipated and not followed in this model

The basic DEBkiss model is described in detail elsewhere (Jager et al. 2013; Jager 2015), including the differences with DEB theory. We showed earlier (Jager et al. 2015) that this model could capture growth and development of *C. sinicus*, from egg to final moult, with only two modifications: accounting for shape differences between nauplii and copepodites, and a complete stop of growth after the final moult to the adult stage (observation 1 in Table 1; a size threshold was used to trigger the final moult). As data on storage or reproduction rates under controlled conditions were not available, the allocation fraction to soma ($\kappa$, see Fig. 1) was rather arbitrarily fixed to a value of 0.8, and model fitting focussed on structural body size. Here we depart from this model, but require additional modifications to capture the other observations in Table 1. The expanded model is schematically shown in Fig. 1, model equations are provided in Table 2, and symbols are explained in Table 3.

**Table 2:** Specification of the DEBkiss model used in this study

Fluxes	Model formulation (mg/day)
Assimilation (for adults: $L=L_{\rm a}$)	$J_{\rm A}= f J_{\rm Am}^{\rm a} L^2$
Somatic maintenance (for adults: $L=L_{\rm a}$)	$J_{\rm M} = J_{\rm M}^{\rm v} L^3$
Maturity maintenance (for adults: $L=L_{\rm a}$)	$J_{\rm J} = J_{\rm J}^{\rm v} \min (L^3,L_{\rm p}^3)$
Growth (only embryos, juveniles and sub-adults)	$J_{\rm V}=y_{\rm AV}(\kappa J_{\rm A}-J_{\rm M})$
Maturation (only embryos and juveniles)	$J_{\rm H} = (1-\kappa )J_{\rm A}-J_{\rm J}$
Storage (only sub-adults)	$J_{\rm R} = (1-\kappa )J_{\rm A}-J_{\rm J}$
Egg production (only adults, option A Fig. 1)	$J_{\rm B}=y_{\rm BA} (J_{\rm A} -J_{\rm M} - J_{\rm J})$

State variables	Model formulation (mg/day)
Structural body mass	$\frac{\rm d}{\rm dt}W_{\rm V}=J_{\rm V}$ with $W_{\rm V}(0)=d_{\rm V} L_0^3$
Assimilate buffer in egg (embryos)	$\frac{\rm d}{\rm dt}W_{\rm B}=-J_{\rm A}$ with $W_{\rm B}(0)=W_{\rm B0}$
Reproduction buffer (sub-adults)	$\frac{\rm d}{\rm dt}W_{\rm R}=J_{\rm R}$ with $W_{\rm R}(0)=0$

Conversions	
Volumetric length (*L*) to dry weight	$W_{\rm V}=d_{\rm V} L^3$
Physical length ($L_w$) to volumetric length	$L = L_{\rm w} \delta _{\rm M}$
Temperature effect on rate constants	$F_{\rm T} = \exp \left( \frac{T_{\rm A}}{T_{\rm ref}} - \frac{T_{\rm A}}{T} \right)$

Symbols explained in Table 3. Note that for embryos (eggs, N1 and N2) $f=1$ in all treatments, and before puberty, $J_{\rm A}$ is multiplied by $\delta$ (see Fig. 1)

**Table 3:** Model parameters and conversion factors used in this study, which are fixed as a constant (C) or default (D) value, or fitted (F) on the data in Figs. 3 and 4

Symbol	Explanation	Value (CI) unit (status)
$d_{\rm C}$	Carbon weight per dry weight (structure)	0.40 mg/mg (C)
$d_{\rm N}$	Nitrogen weight per dry weight (structure)	0.10 mg/mg (C)
$d_{\rm V}$	Dry weight per body volume (structure)	0.27 mg/mm^3^ (C)
*f*	Scaled functional response	
	–Maximum food treatment	1 [–] (C)
	–Medium food treatment	0.666 (0.651–0.678) [–] (F)
	–Low food treatment	0.548 (0.535–0.561) [–] (F)
$J_{\rm Am}^{\rm a}$	Maximum area-specific assimilation rate	0.0852 (0.0832–0.0870) mg/mm^2^/day (F)
$J_{\rm J}^{\rm v}$	Volume-specific maturity maintenance rate	0.268 (0.206–0.281) mg/mm^3^/day (F)
$J_{\rm M}^{\rm v}$	Volume-specific somatic maintenance rate	0.0106 (0.00982–0.0113) mg/mm^3^/day (F)
$L_{0}$	Volumetric length, start development	0.01 mm (D)
$L_{\rm p}$	Volumetric length, puberty (juv. to sub-adult)	0.303 (0.295–0.308) mm (F)
$L_{\rm a}$	Volumetric length, adulthood (final size)	
	–Maximum food, temperature 4 °C	1.07 (1.05–1.09) mm (F)
	–Maximum food, temperature 8 °C	1.05 (1.02–1.08) mm (F)
	–Maximum food, temperature 12 °C	0.985 (0.953–1.02) mm (F)
	–Medium food, temperature 8 °C	0.794 (0.771–0.819) mm (F)
	–Low food, temperature 8 °C	0.629 (0.602–0.656) mm (F)
$T_{\rm A}$	Arrhenius temperature	8200 (8020–8380) K (F)
$T_{\rm ref}$	Reference temperature (10 °C)	283 K (C)
$W_{\rm B0}$	Dry weight of a single egg	0.48 $\upmu$g (C)
$y_{\rm AXc}$	Yield of assimilates on food (carbon)	0.80 mg/mg (D)
$y_{\rm BA}$	Yield of egg buffer on assimilates	0.95 mg/mg (D)
$y_{\rm VA}$	Yield of structure on assimilates	0.80 mg/mg (D)
$\delta$	Factor decrease in assimilation of early stages	0.535 (0.522–0.547) [–] (F)
$\delta _{\rm M}$	Shape correction coefficient (nauplii/cop.)	0.44/0.38 [–] (C)
$\kappa$	Fraction allocation to soma	0.483 (0.464–0.493) [–] (F)

Confidence intervals for fitted parameters are approximate 95% intervals by profiling the likelihood function. All rate constants are referenced to 10  °C. Note that $\delta _{\rm M}$ links volumetric length to total body length of nauplii and prosome length for copepodites

#### Growth

**Fig. 2:**
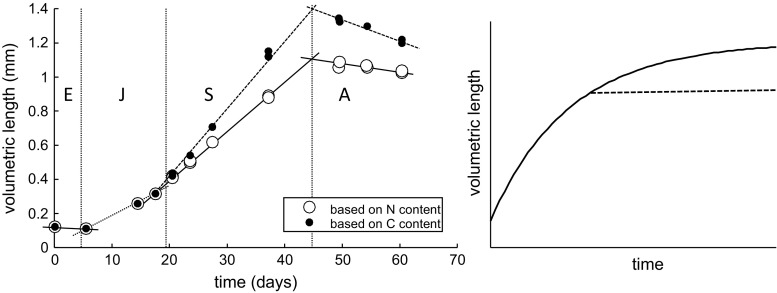
Volumetric body length of *C. finmarchicus*, calculated from N content (representing structural biomass plus egg buffer) and C content (including lipid storage as well) from egg to adulthood (*left panel*) at 8 °C (data from Campbell et al. 2001). Life stages indicated are Embryo, Juvenile, Sub-adult and Adult (Fig. 1). *Straight lines* drawn by eye to indicate linearity. *Right panel* shows a standard (*solid line*) and truncated (*broken line*) von Bertalanffy curve on length basis for reference

The general growth pattern of *C. finmarchicus* is shown in Fig. 2. The length measure used throughout this paper is the volumetric length, i.e., the cubic root of estimated body volume from the measured N and C content. Even though this measure is rather abstract, it has the advantage that nauplii and copepodites can be compared in the same graphs, that C and N growth can be compared, and that the growth patterns can easily be compared to the von Bertalanffy curve as expected for most animals in a DEB context (Nisbet et al. 2000). The volumetric length based on N content represents structural biomass plus the egg buffer for the embryo (including the non-feeding naupliar stages). The length based on C content includes all biomass (egg buffer, structure and lipid storage). As long the C/N ratio is 4 ($d_{\rm C}/d_{\rm N}$ in Table 3), volumetric length based on C and N will be the same; a higher value for C-based length in Fig. 2 indicates lipid storage. The conversion from the three state variable (Table 2, all in dry weight) to volumetric length measures is explained in more detail later.

As with the congeneric *C. sinicus* (Jager et al. 2015), the embryo only decreases in size over time (Fig. 2). Egg buffer is converted into structural biomass, whereby mass is lost on maturation, maintenance and conversions. In contrast to *C. sinicus*, however, the post-embryonic growth of *C. finmarchicus* does not resemble a truncated von Bertalanffy curve (observation 2 in Table 1). Rather, the growth curve for juveniles and sub-adults consists of two more-or-less linear phases (Fig. 2). Such a pattern has also been observed for other calanoid species, such as *C. marshallae* (Peterson 1986) and the genus *Acartia* (Miller et al. 1977). Deviations from the von Bertalanffy curve in the early life stages are quite common, especially among animal species sporting larval development, and several modifications in a DEB context have been proposed (Kooijman 2014). Here we assume that the copepods go through a ‘type A acceleration’, where the specific assimilation rate makes a step-up in one of the late naupliar or early copepodite stages.

#### Lipid storage

Lipid storage starts well before reaching the final size and increases over time, not only in absolute sense but also relative to body structure (observation 5, and Harris et al. 2000). The initiation of storage, however, does not come at the expense of structural growth (observation 6). Therefore, we propose to view the storage as a compartment in the $1-\kappa$ branch, and thus as a ‘reproduction buffer’ in the DEB context. In this way, the investment in lipid storage is decoupled from the growth process, and lipids will accumulate over time.

The main difference between DEB theory and DEBkiss is that the latter does not use a reserve compartment in between feeding and the energy-requiring processes. It is tempting to consider the lipid sac as such a reserve, but the reserve of DEB theory will not capture observation 5 and 8, at least not without major modification of reserve dynamics. In DEB theory, under constant food availability, the ‘weak homeostasis’ assumption leads to a constant ratio of reserve versus structural mass. This translates into a constant C/N ratio over ontogeny, contrary to the observations on *C. finmarchicus* (Figs. 2, 5). In an adaptation of the standard DEB model to insects (Llandres et al. 2015), facing a similar challenge, the same choice was made as in the present study: to include a reproduction buffer, building up over the larval stages.

In DEBkiss, following DEB theory, the $1-\kappa$ branch is used for maturation up to ‘puberty’, after which the flux is switched to the reproduction buffer (see Fig. 1). As no eggs are produced before the final adult stage, we refer to these post-puberty individuals as sub-adults. Maturity maintenance is also paid from the $1-\kappa$ branch, and takes precedence over maturation or storage. Note that maturity maintenance does not increase anymore after puberty (see Table 2), as the investment in maturity itself has ceased. The start of lipid storage seems to coincide with the step-up in the growth rate (Fig. 2). To limit the number of switches in the model, we therefore propose a single switch (for ‘puberty’) triggering both events.

#### Adult stage

After reaching adulthood, the lipid storage decreases, and there also seems to be a slight decreasing trend in structural biomass (Fig. 2, observation 8). In this study, we will not attempt to include the bioenergetic aspects of the early adult stage into the model. We also do not provide a mechanistic explanation for the changes in maximum size with temperature and food level (observations 3 and 4 in Table 1), but resort to a more descriptive approach, taking maximum body length as a free parameter at each temperature.

As the adults do not grow (and do not reach their predicted asymptotic maximum size), the $\kappa$-rule allocation cannot work for them. In line with the previous work on *C. sinicus* (Jager et al. 2015), we make the assumption that all of the assimilated energy, minus maintenance costs, is used for egg production (option A in Fig. 1). As an alternative, we consider that somatic maintenance costs are paid from the lipid storage (option B). These options should be regarded as preliminary.

The energetics of the adult stage will be addressed in more detail in the discussion.

#### Effects of food and temperature

An increase in temperature is assumed to increase all rate constants (parameters with day$^{-1}$ in their unit) by the same factor according to the Arrhenius relationship (Table 2, and see Jager et al. 2015). A change in temperature thus effectively stretches the time axis on the growth curves. Food limitation affects the scaled functional response (*f*, Table 2 and 3). Both temperature and food limitation also affect the length at adulthood ($L_{\rm a}$), but none of the other parameters.

### Model optimisation

All calculations were performed in Matlab R2016a. Maximum-likelihood optimisation was performed, assuming independent and normally distributed errors after square-root-transformation. Confidence intervals were calculated by profiling the likelihood function (see Jager and Zimmer 2012, for statistical details).

#### Data on carbon and nitrogen content

For model calibration, we used the data for C and N content over time from Campbell et al. (2001). The N content ($W_{\rm N}$) is used as a proxy for structural biomass, and the C content ($W_{\rm C}$) for structural biomass plus lipid storage. These data are translated into corresponding volumetric lengths ($L_{\rm N}$ and $L_{\rm C}$) using the conversion factors in Table 3:

1 $$L_{\rm N} = \left( \frac{W_{\rm N}}{d_{\rm N} d_{\rm V}} \right) ^{1/3},\quad L_{\rm C} = \left( \frac{W_{\rm C}}{d_{\rm C} d_{\rm V}} \right) ^{1/3}$$

For the embryo stage, the resulting estimates of body length will include the egg buffer. The volumetric length from the N content is thus compared to a model estimate combining structural body mass ($W_{\rm V}$) and egg buffer ($W_{\rm B}$). Volumetric length from the C content is compared to a model estimate using all body weight components:

2 $$L_{\rm N} = \left( \frac{W_{\rm V}+W_{\rm B}}{d_{\rm V}} \right) ^{1/3},\quad L_{\rm C} = \left( \frac{W_{\rm V}+W_{\rm B}+W_{\rm R}}{d_{\rm V}} \right) ^{1/3}$$

In the translations in Eqs. (1) and (2), the dry-weight density ($d_{\rm V}$) and carbon content ($d_{\rm C}$) of structure are used. In reality, storage lipids will have a higher carbon content and lower water content than structure and hence contributes less to the total volume of the animal. Using the properties of structure helps to clarify the patterns in the data and facilitates the calculations as it avoids the introduction of new conversion factors for the storage compartment. The total dry weight of the individual can still be derived in the model as $W_{\rm V}+W_{\rm B}+W_{\rm R}$ (although more detail might be needed to deal with differences in composition of these compartments), and the physical length can still be calculated from $W_{\rm V}$ (see Table 2).

We do not use all of the measurements on C content for adults as C content decreased after reaching adulthood (especially at 8 and 12 °C, observation 8). As explained earlier, we do not include a dynamic model formulation for the energetic aspects of the early adult stage (removed data shown in supp. info.).

#### Data on respiration

The data on C and N content contain insufficient information to estimate the specific somatic maintenance rate ($J_{\rm M}^{\rm v}$). This parameter is mainly determined by the curvature of the growth curve on a length basis, and, as shown in Fig. 2, the growth of the copepodites is almost linear over time. To avoid unrealistic estimates of the maintenance rate, we include measurements for the respiration rate versus temperature for C5 of *C. finmarchicus* (Clarke and Bonnet 1939) as additional data set into the optimisation procedure. We assume that respiration is dominated by the somatic maintenance costs, as the animals were kept for a day without food prior to the measurements. We assume that starvation leads to a halt of the other energy-requiring processes, including maturity maintenance (following Kooijman 2010, p. 50, 118). A carbon-based respiration rate (in mg C/day) for each temperature can then be calculated as:

3 $$J_{\rm Dc} = J_{\rm M}^{\rm v} L^3 F_{\rm T} d_{\rm C}$$

A representative volumetric length (*L*) for the respiration data was estimated from the N content of C5 at 8 °C (Campbell et al. 2001). The carbon-based respiration rate was translated to oxygen use by assuming a respiration quotient of 0.8.

### Model predictions

Once the model parameters are established, they can be used to predict life-history traits that were not used for fitting. We can easily compare the C/N ratio ($F_{\rm CN}$) as reported by Campbell et al. (2001) to the values predicted by the model:

4 $$F_{\rm CN} = \frac{d_{\rm C}(W_{\rm V}+W_{\rm B}+W_{\rm R})}{d_{\rm N}(W_{\rm V}+W_{\rm B})}$$

The C/N ratio allows us to judge whether the model is able to predict a reasonable ontogenetic pattern of lipid build-up.

The proposed model deals with all of the four life stages in Fig. 1. There are therefore three life-cycle switches: birth (start of feeding), puberty (increase in feeding rate and start of storage), and adulthood. Clearly, birth ($W_{\rm B}=0$) should coincide with the start of the first feeding stage (N3), and adulthood ($L=L_{\rm a}$) with the final moult to adults (C6). For puberty ($L=L_{\rm p}$), the switch should fall in one of the early copepodite stages, given observation 2 and 5 in Table 1. We compare model predictions for the age at these switching events to the mean age at the start of various naupliar and copepodite stages as determined by Campbell et al. (2001).

To provide a reality check on the model and its parameterisation, we compare the assimilation rates from the model to values for filtration and feeding rates as a function of body size from the literature (Marshall and Orr 1956; Meyer et al. 2002). The assimilation flux in the model ($J_{\rm A}$, Table 2) can be translated into a carbon-based feeding rate (in mg C/day) as follows:

5 $$J_{\rm Xc} = d_{\rm C} J_{\rm A} / y_{\rm AXc}$$

As the assimilation flux is stepped-up at puberty (the factor $\delta$ in Fig. 1), so will the predicted feeding rate from the model. For the filtration rates, we can only make a qualitative comparison, as the proportionality constant between filtration volume and feeding rate is unknown for the conditions in the experimental test (Marshall and Orr 1956).

Båmstedt et al. (1999) report egg production rates (in mg C/day) at various ingestion rates (also in mg C/day). The ingestion rate is given by Eq. (5), and the reproduction rate can be predicted by assuming that only food is used to fuel maintenance costs and egg production (option A in Fig. 1; Table 2). This mass flux can be translated to a carbon flux using $d_{\rm C}$. It should be stressed that this calculation ignores the potential contribution of lipid storage and can therefore only be used to illustrate the potential of feeding to fuel reproduction and maintenance. As an alternative (option B in Fig. 1), we also make a prediction assuming that somatic maintenance costs are paid from the lipid storage. Additionally, we use the data for egg production rate versus body length of Rey et al. (1999) for a comparison to model predictions.

## Results

### Model fits and their consequences

**Fig. 3:**
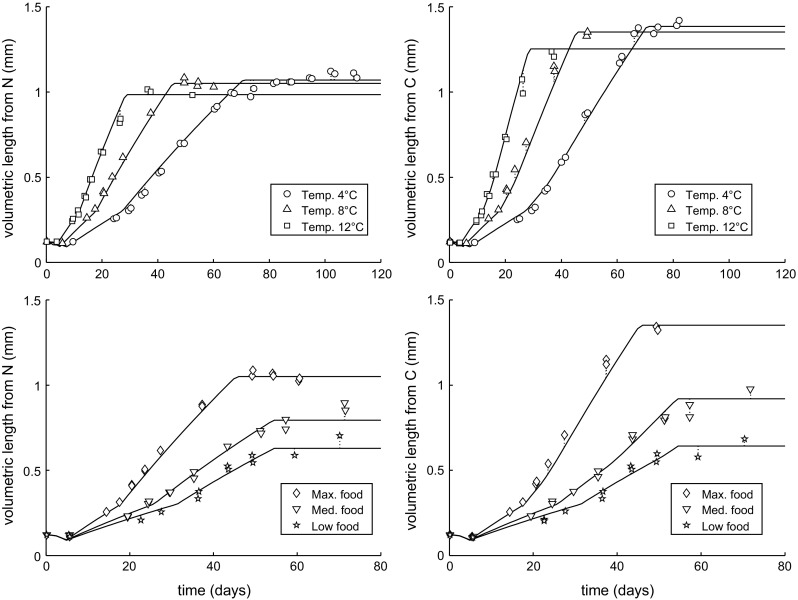
Fit of the model to the data for growth of *C. finmarchicus* at various temperatures (*top*) and food levels (*bottom*). Measured data for C and N content (Campbell et al. 2001) have been translated to volumetric length (conversion factors in Table 3). *Left panels* length based on N content (structure plus egg buffer). *Right panels* length based on carbon content (structure, egg buffer, and lipid storage)

**Fig. 4:**
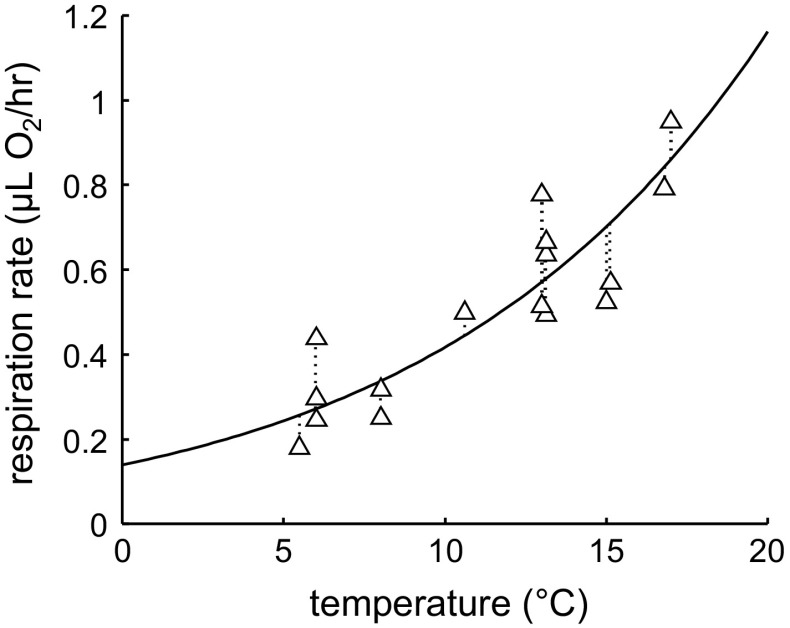
Fit of the model to the data for respiration rate in C5 of *C. finmarchicus* at various temperatures. Measured data from Clarke and Bonnet (1939) fitted simultaneously with the body length data in Fig. 3

The modified DEBkiss model was fitted to the experimental data for N and C content at different temperatures and food levels of Campbell et al. (2001) (Fig. 3), and the respiration data for C5 from Clarke and Bonnet (1939) (Fig. 4). The model provided a good fit to all data sets simultaneously using 14 fitted parameters (Table 3), which, given the amount of data in Figs. 3 and 4, is quite reasonable. The inclusion of the respiration data ensured that the specific maintenance rate ($J_{\rm M}^{\rm v}$) could be identified with a tight confidence interval. The same Arrhenius temperature governs the temperature effect on the growth pattern as well as on the respiration rates. As both processes were well captured, this supports the use of one temperature correction for all rate constants. The response to limiting food levels was included as a different value for the scaled functional response (*f*) at each food level. Note that $f=1$ marks ad libitum feeding (fixed for the high-food treatment) and $f=0$ would mark complete starvation.

**Fig. 5:**
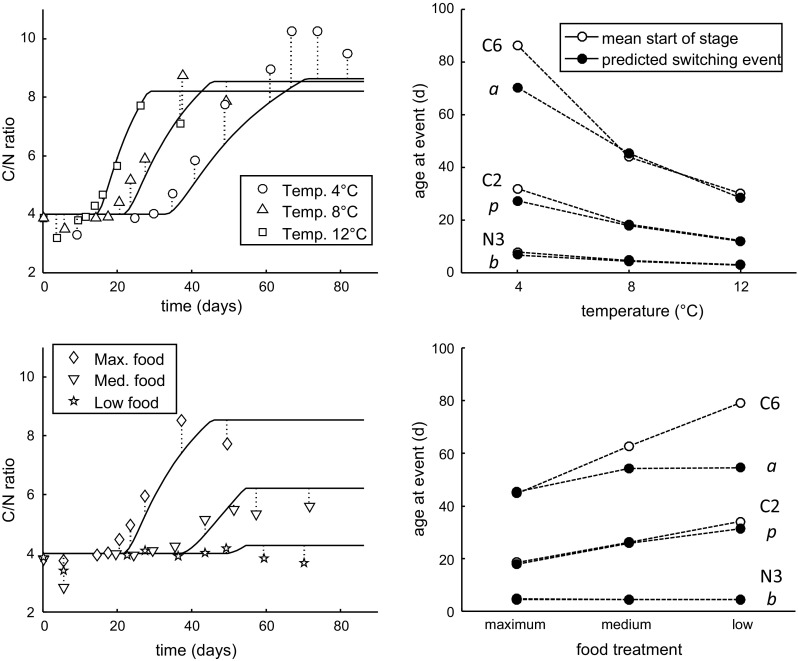
Consequences of the model fits in Figs. 3 and 4. *Left panel* comparison of predicted and reported (Campbell et al. 2001) C/N ratios with age. *Right panel* comparison of the timing of the three switching events in the model (birth, puberty and adulthood, see Fig. 1) to the observed mean start of various life stages (N3, C2 and C6) (Campbell et al. 2001)

Figure 5 shows the consequences of the fitted model parameters for the C/N ratio and the age at the three switches in the life cycle (birth, puberty and adulthood, see Fig. 1). The C/N ratio does not provide additional information to the length measures based on C and N content used in the model fit (Fig. 3), but allows for a more straightforward interpretation of the model’s ability to predict realistic build-up of lipid storage (as lipid accumulation will only increase the C content of the body). The pattern of lipid storage was captured quite well by the model. At the lowest test temperature, the C/N ratio was, however, consistently higher than predicted over the phase of lipid build-up (which was not obvious from the fits in Fig. 3). The reasons for this deviation are unclear, but it might well be a phenotypic adaptation to cold environments, whereby some model parameters are affected by temperature in a slightly different way than currently assumed.

The reduced lipid build-up at limiting food levels was well captured by the model. The ability to reproduce this pattern depended almost entirely on maturity maintenance and thus on the parameter $J_{\rm J}^{\rm v}$. Maturity maintenance holds primacy in the $1-\kappa$ branch; these costs are paid first, and the remaining assimilates can be accumulated as storage. If maturity maintenance costs are small, there would still be substantial accumulation of lipids at limiting food levels; less energy from food is available for storage, but since growth is also inhibited, the C/N ratio would still increase similarly to the animals at high food. The value for $J_{\rm J}^{\rm v}$ that we fitted is very high. In a DEB context, a default value is often applied based on the specific somatic maintenance rate ($J_{\rm M}^{\rm v}$) and $\kappa$ (Jager 2015; Kooijman 2010, p. 50), which, given our value for $\kappa$, would predict a value for $J_{\rm J}^{\rm v}$ much closer to that of $J_{\rm M}^{\rm v}$. As the value for $J_{\rm J}^{\rm v}$ was only fixed by the behaviour of the lipid storage at low food levels, we should consider alternative explanations. For example, food-limited individuals may increase $\kappa$ to sustain somatic growth at the expense of storage, or perhaps maturation does not cease completely at puberty but competes with lipid storage. These alternatives, however, require larger deviations from the basic DEBkiss model, require more parameters to be fitted to the data, and therefore more detailed experimental work.

The DEBkiss model includes a representation of embryonic development (Fig. 1; Table 2) and predicts the initiation of feeding (birth) when the energy provided by the egg buffer is depleted ($W_{\rm B}=0$). The point of birth, as estimated by the model, closely corresponded to the start of the N3 stage in all treatments, which is the first feeding stage in *C. finmarchicus* (Fig. 5, open symbols are hidden underneath the closed ones). At puberty, assimilation switches to a higher value and allocation to lipid storage starts. The predicted switch for puberty coincided with the start of stage C2 (Fig. 5). Even though the moult from N6 to C1 represents a huge change in morphology, the metabolic change appears to occur slightly later. However, it should be noted that, in the model, a sudden switch was implemented for a process that will likely be more gradual in reality.

The switch to adulthood, as predicted by the model, corresponded well with the mean start of the adult stage for the highest two temperatures and the high-food treatment (Fig. 5). For the lowest test temperature and the limited food treatments, the model underpredicted the age for the start of the adult stage. One possible explanation is a limitation in the data set: at lower temperatures and limiting food levels, the cohorts became less synchronised with age (Campbell et al. 2001). For example, in the 4 °C cohort, animals became adult somewhere between age 70 and 110 days; the transition thus smeared out over some 40 days. At the lowest food treatment, adults started to appear at age 60 days, even though the mean start of adulthood was estimated at 80 days. The average C and N content within such a variable cohort should be interpreted with care.

### Model predictions for ingestion and reproduction

The comparison of predicted to measured feeding rates consists of a qualitative and a quantitative element. Qualitatively, we want to check the validity of the predicted pattern of ingestion rate versus body size; the model predicts that feeding rate scales with body length squared (Table 2) with a step-up in the feeding rate (corresponding to the factor $\delta$ in Table 3) at puberty. Quantitatively, we need to check whether observed feeding rates match the predicted ones as this provides an essential check on the energy budget.

**Fig. 6:**
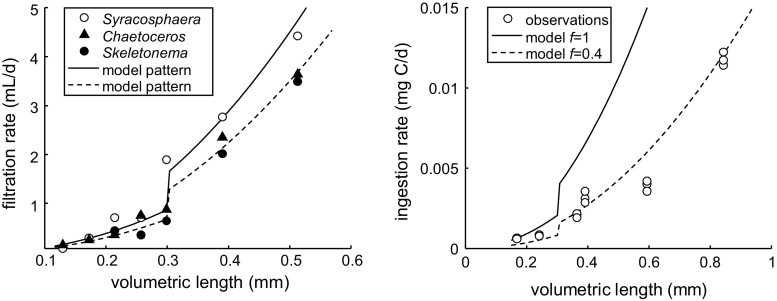
*Left panel* filtration rates for different food sources versus volumetric length (stage N3–C3) at unknown temperature (Marshall and Orr 1956). Model predictions assuming reduced feeding rates (factor $\delta$ from Table 3) until ‘puberty’. Specific filtration rates set to 14 and 18 mL/mm$^2$/day for the low and high prediction, respectively, for an approximate fit. *Right panel* measured ingestion rates (Meyer et al. 2002) versus volumetric length (calculated from reported C content, for stage N4/5-adult). Model predictions using two values for the scaled functional response, the lower estimate providing an approximate fit to the data

Qualitative support for a step-up in feeding rate could be obtained from the work of Marshall and Orr (1956). These authors measured filtration rates of *C. finmarchicus* (stage N3 to C3) on different algal foods (Fig. 6, left panel). Body length was estimated from the N content at each stage (Campbell et al. 2001). Filtration rates should be proportional to ingestion rates (at constant food concentrations), and the latter follows from the model (Eq. 5). Figure 6 shows that the predicted step-up pattern was quite consistent with the observed filtration rates, with *Syracosphaera* leading to somewhat higher rates than the other two food types. It should be noted that these predictions are not fits; we do not know the proportionality between filtration rates and feeding rates, and the temperature at which the experiments were performed was not reported. However, we did use the fitted value for $\delta$ for the step-up pattern, showing that the degree of change in assimilation needed to fit the growth data matches the observed change in filtration rate.

The right panel of Fig. 6 shows ingestion rates as reported by Meyer et al. (2002). Body length was estimated from the C content provided by the authors and may be biassed by lipid storage. However, comparing the C content at the various stages to the N and C values reported by Campbell et al. (2001), it is most likely that the animals in the feeding experiments did not build up a lipid storage. The food level in the feeding experiments was also much lower than in the high-food treatments for the growth experiments (nominally, 120 vs. 500 $\upmu$g C/L). The step-up pattern was not obvious from this data set, although the general pattern of feeding scaling with length squared held (as expected from the DEBkiss model). The observed ingestion rates were lower than expected (corresponding to $f=0.4$), which is consistent with the low carbon contents and likely reflects the low food level provided.

Paffenhöfer (1971) performed detailed feeding studies with a related copepod species, *C. helgolandicus*. These data were generally consistent with the model predictions, although they seem to indicate a more gradual increase in feeding rates over the early copepodite stages (see. supp. info.).

**Fig. 7:**
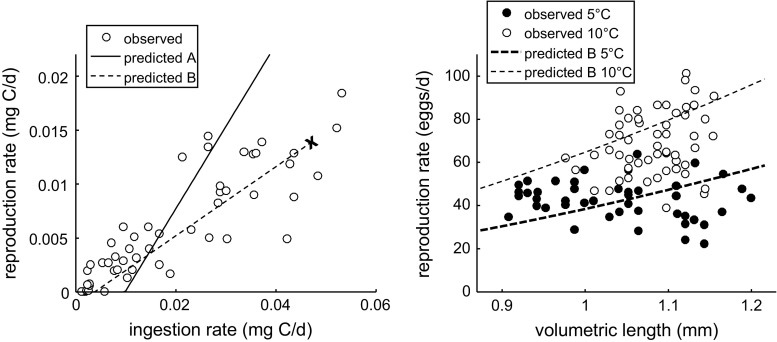
*Left panel* reproduction rate versus ingestion rate; data from Båmstedt et al. (1999). Two predictions are shown, option A and option B (with increased egg costs, see text) as explained in Fig. 1. The *cross * marks the highest feeding and reproduction rates predicted from the model. *Right panel* reproduction rates versus body length at two temperatures; data from Rey et al. (1999), and only model predictions for option B are plotted

Båmstedt et al. (1999) reported reproduction rates at a range of ingestion rates (Fig. 7). These authors included food levels high enough to saturate the ingestion rate, and the maximum ingestion rate predicted by the model (cross mark in Fig. 7) corresponded quite well to the highest observed ingestion rates (which were indeed much higher than the adult values shown in the right panel of Fig. 6). The maximum ingestion rate from the model was consistent with the observations on this endpoint at high food availability.

The observations on reproduction and ingestion rates revealed a linear relationship (Fig. 7, left panel): approximately 30% of the ingested carbon was excreted with eggs across all ingestion rates. In the ‘option A’ topology for adults (Fig. 1), reproduction is paid from ingestion, after somatic and maturity maintenance costs have been subtracted (see Table 2). The relationship predicted from this model formulation consists of two linear phases: below a certain ingestion rate, all of the assimilated energy is needed to cover maintenance needs. At low food levels, the model underestimated reproduction, whereas it overestimates at the high food levels. We could obtain a closer match from an alternative allocation scheme (option B in Fig. 1), where somatic maintenance costs are paid from the lipid storage, and adding an ad-hoc increase in the egg costs (lowering the yield to $y_{\rm BA}=0.4$). Option B, with the lowered yield, was also consistent with the reproduction rates versus body length reported by Rey et al. (1999) (Fig. 7, right panel). The increase in reproduction rate both with temperature and (to a lesser extent) with body size was consistent with the observations.

## Discussion

### Model evaluation: growth pattern

From the standard DEBkiss model, we would expect growth to follow the von Bertalanffy curve, as long as environmental conditions are constant, and indeed this growth pattern is very common among animals (Nisbet et al. 2000; Kooijman 2010, p. 52). Copepod growth, however, deviates from that expectation and is often describes as exponential (on weight basis, see e.g., Peterson 1986; Miller et al. 1977; Escribano and McLaren 1992). In the DEB context, we need a mechanistic explanation for this deviating growth pattern, as a modification of the general pattern for animals.

Copepods stop growth after the final moult, well before reaching their asymptotic size. This implies that the von Bertalanffy curve is truncated, yielding a close-to-linear relationship for body length (Fig. 2). The von Bertalanffy curve follows from the model as the assimilation flux ($J_{\rm A}$) scales with a surface area, and the maintenance costs ($J_{\rm M}$) with a volume (Table 2), while $\kappa$ remains constant (Jager et al. 2013). The (truncated) von Bertalanffy curve fitted well for the feeding stages of *C. sinicus* (Jager et al. 2015), but the growth curve for *C. finmarchicus* differs from this expectation and reveals two phases of (almost) linear growth (Figs. 2, 3). As a parsimonious solution, we introduced a simple step-up in the assimilation rate at puberty to match the growth pattern of *C. finmarchicus*. Support for this step-up is offered by the filtration rate measurements in Fig. 6. Why a similar adaptation was not needed for *C. sinicus* is unclear; it may relate to the species or to the experimental conditions (e.g., the type of food offered).

### Model evaluation: maximum size

In the current model, maximum size is treated as a model parameter that takes on different values depending on temperature and food level. This deviates from the approach taken in DEBkiss and DEB theory where maximum body size is explained from underlying processes: growth ceases when all of the assimilated energy allocated towards growth and somatic maintenance (the $\kappa$ branch in Fig. 1) is used for the latter. The asymptotic volumetric length relates to the model parameters as follows:

6 $$L_{\infty } = f \frac{\kappa J_{\rm Am}^{\rm a}}{J_{\rm M}^{\rm v}}$$

Food limitation will decrease *f* and thus decreases the ultimate body size. Temperature affects both rate constants by the same factor and hence has no effect on ultimate size. These simple rules do not apply for copepods as these animals do not reach their final size asymptotically: growth ceases rather abruptly after the final moult. Ultimate size in these species thus cannot be interpreted as a balance between a source (assimilation) and a sink (maintenance), but is best viewed as a switching event to a different allocation scheme (Fig. 1).

Within DEB theory, life-stage switching is assumed to be triggered by the state of maturity (Augustine et al. 2011), which, under certain conditions, yields stage transitions at constant body size. In DEBkiss, investment in maturity is included (the mass flux $J_{\rm H}$ in Fig. 1) but not followed as a state variable. We could easily add this state variable to our model, but this would not help for switching events after puberty. At puberty, the investment in maturation is assumed to be switched completely to the lipid storage, which implies no further increase in the state of maturity.

Interestingly, maximum size of *C. finmarchicus* increases with decreasing temperature (Table 3). This seems to be a general trend in ectotherms, and the suggested explanations often include arguments based on the von Bertalanffy curve (Atkinson and Sibly 1997; Perrin 1995). As discussed above, explanations based on Eq. (6) cannot be invoked for species with determinate growth. A more promising hypothesis is that size at moulting may be determined by oxygen supply (as was indicated for insects, Callier and Nijhout 2011), rather than by the energy budget per se. As oxygen demand and supply may respond differently to temperature, different adult sizes may result in species with a final moult (the potential role of oxygen is also discussed in Atkinson and Sibly 1997).

However, we should also keep open the option that the effect of temperature on maximum size is an experimental artefact. In the experiments of Campbell et al. (2001), the actual mean food concentrations were substantially lower than the nominal ones and showed a decrease with increasing temperature. At low temperatures, feeding rates are lower, and it may be easier to maintain relatively high food concentrations in an experimental situation. The effect of temperature on maximum size may thus be partly caused by a different effective food concentration.

Maximum size of *C. finmarchicus* decreased with decreasing food concentration (Table 3, and observation 4 in Table 1). This is a common response to food limitation (e.g., Jager et al. 2013), but, again, the explanation offered in Eq. (6) does not hold for copepods. For insects, which also exhibit true determinate growth, moulting seems to be initiated at a certain size where oxygen supply falls short of the demand (Callier and Nijhout 2011). However, the same authors also point at the existence of a size-independent mechanism triggering moulting: when growth is inhibited (for example by starvation), larvae will moult before attaining the critical body size.

Owing to these complications, we decided to use maximum length as a model parameter, whose value depends on temperature and food availability. Given the available data, there does not seem to be a simple way, consistent with DEB theory, to explain maximum size in copepods and how it is affected by food and temperature.

### Allocation in the adult stage: maturation and diapause

The energetics for the adult stage deviates from that in standard DEB-based models for animals. As growth stops after the final moult (well before approaching the asymptotic size), the $\kappa$-rule allocation cannot continue to operate in adults (otherwise, growth would continue). For this reason, we propose a simpler scheme for adult energetics, removing the $\kappa$ split of the assimilation flux, and only considering maintenance processes and reproduction as energy sinks (see Fig. 1). A comparable switch in allocation scheme was proposed for holometabolous insects by Llandres et al. (2015).

The transition to adulthood seems to be accompanied by substantial energetic costs as the lipid content declines after the final moult (observation 8 in Table 1). These costs likely represent gonad maturation, which was estimated to require some 70 $\upmu$g C in *C. finmarchicus* (Rey-Rassat et al. 2002). We did not include the process of gonad maturation into the model at this point and therefore also excluded the data points for C content in the adult phase that show the decline in lipids (see supp. info.). The current data set has insufficient information on this process as we only have mean body compositions, and the synchronisation of the experimental cohort has become quite poor at the start of the adult stage (Campbell et al. 2001).

We can, however, look at the conceptual model of Rey et al. (1999) for an outline of a future model adaptation. These authors estimate that, at 10 °C, it takes 6 days between the final moult and the production of the first clutch. In this scheme, the authors suggest that, during most of this time, the animals do not feed but use their lipid supply to fuel their maintenance needs (including the costs of mating) and maturation of the gonads. The last part of gonad maturation, and the preparation of the first clutch of eggs, seems to require feeding. After the first clutch, the reproduction rate gradually increases, along with the ingestion rate, reaching an optimum after some 11 days post-moulting.

We can compare this scheme to some of the bioenergetic constraints following from our parameterised model. The model predicts that some 140 $\upmu$g of C in lipid storage is built up at the end of the C5 stage (at 8 °C). Somatic maintenance costs of the adults will be some 5 $\upmu$g C per day (at 10 °C), and thus 30 $\upmu$g over 6 days. Rey-Rassat et al. (2002) estimated that 70 $\upmu$g C was lost in females during maturation of the gonads. Our calculation suggests that this amount needs to be broken up in 30 $\upmu$g for somatic maintenance and 40 $\upmu$g for maturation (and the moulting process). In the laboratory setting, some 70 $\upmu$g of C should be remaining in the lipid storage after gonad maturation. In a field situation, this is the maximum amount of carbon that could thus be used for overwintering, while still allowing for gonad maturation. At 10 °C, this remaining storage amount would only cover two weeks of somatic maintenance costs in absence of feeding. However, during overwintering, maintenance costs will be strongly reduced (Hirche 1983). This calculation illustrates how our model can be used to elucidate the energetic constraints on diapause and maturation.

### Allocation in the adult stage: reproduction

Whatever remains in the lipid storage after gonad maturation could be used to complement egg production, or to fuel somatic maintenance. Our option B (Fig. 1) follows the latter assumption. This is consistent with the decrease in lipid sac volume observed in females over a period of reproduction (Rey et al. 1999; Plourde and Runge 1993). Males also seem to rely heavily on their lipid storage to sustain their maintenance needs with limited feeding (Irigoien et al. 2000; Paffenhöfer 1971). Some studies show, however, that lipid storage is used to complement egg production. Hirche et al. (1997) showed higher reproduction rates (over a range of food availabilities) for previously fed in contrast to previously starved females. Even in the absence of food, previously fed animals were able to sustain egg production (contrasting the data shown in Fig. 7). Furthermore, egg production can also be (partly) fuelled from structural body components (Mayor et al. 2009). Adult females will obviously obey the conservation laws for mass and energy, but the rules by which they use the energy available from food, storage and structure remain unclear and may very well depend on environmental and internal cues (see also discussion in Harris et al. 2000).

To obtain a good correspondence to the observed reproduction rates (Fig. 7), we had to increase the overhead costs for egg production (decrease the yield $y_{{\rm BA}}$). It is difficult to explain why $y_{\rm BA}$ should be 0.4 instead of the default of 0.95 that works well in most DEB and DEBkiss applications. Also for *C. sinicus* (Jager et al. 2015), the default $y_{{\rm BA}}$ produced very reasonable reproduction rates. In that study, however, $\kappa$ was fixed to a default of 0.8, which may have biassed the reproduction estimates. The default for $y_{{\rm BA}}$ is not based on strong empirical or theoretical considerations, but rather on the assumption that reproduction is mainly the encapsulation of assimilates into eggs, which should not be accompanied by a large amount of metabolic work. This assumption is, however, not supported by our current analysis, and especially the left panel of Fig. 7 is telling: only 30% of the ingested carbon is converted into eggs, even at abundant food. For *A. tonsa*, a similar linear relationship between ingestion and reproduction was demonstrated (Kiørboe et al. 1985), with 36% of the ingested carbon excreted as eggs. At abundant food, maintenance costs play a relatively small role in the total budget, and the conversion of food to eggs is dominated by the two yield coefficients $y_{\rm AX}$ and $y_{\rm BA}$. As the maximum ingestion rate observed is close to the predicted one, it is unlikely that $y_{\rm AX}$ is too far off, leaving $y_{\rm BA}$ as the prime candidate for modification.

The discussion above focusses on carbon, whereas an egg has to contain a balanced set of compounds (specific lipids, proteins, etc.) to allow successful development to the first feeding naupliar stage. It is reasonable to assume that egg production places higher demands on food quality than maintenance processes, and the low conversion of carbon from food to eggs may thus relate to stoichiometric constraints. For example, carbon in food may be largely excreted as the organism requires specific compounds from the food that are in short supply, or the organism has to use carbon for the metabolic work needed to convert compounds in the food to the specific form needed by the embryo. Solving these issues requires more detailed study, but in the meantime, we suggest using a lower value of $y_{\rm BA}$ for *C. finmarchicus* than the default in Table 3.

## Conclusion

The DEBkiss model was able to explain the life-history data for *C. finmarchicus* well with only a few modest modifications of this generic framework. The typical growth pattern of this species was explained by a switch in energy allocation at adulthood (when investment in growth ceases), and a switch increasing the maximum specific assimilation rate ($J_{\rm Am}^{\rm a}$) at puberty (corresponding to the start of the C2 stage). Such an acceleration of metabolism early in life is often included in DEB models as well (Kooijman 2014), and seems to be a common feature in copepod growth curves, although it was not observed in our previous study with *C. sinicus* (comparison between both species in supp. info.). The build-up of lipid storage was not covered in our previous work, but we show here that this process can be captured by treating this storage as a reproduction buffer (Fig. 1). The resulting parameter set provides a good fit to the data for C and N content over the life cycle for different temperature and food treatments, as well as for the available respiration data. The parameter set also results in reasonable patterns for ingestion and reproduction rates, although the data suggest higher overhead costs for egg production than initially assumed. Providing a coherent picture of bioenergetics under constant laboratory conditions is a necessary first step, so that the model can subsequently be applied to interpret the effects of stressors (e.g., toxicants) and to predict life history under realistic (time-varying) conditions.

However, some fundamental questions remain. Even though lipid storage was described well, very high maturity maintenance costs were needed to catch the pattern that less storage is built up at limiting food levels. Alternative explanations should be investigated with more detailed data sets. We did not explicitly deal with diapause in this study, although the energetic constraints were established. The critical issues in including this part of the life cycle will be the establishment of the effective maintenance rate coefficient (which will be lower than during feeding periods) and the cues to enter and end diapause. Another remaining question concerns the mechanism(s) for the changes in maximum body size with temperature and food level. The standard explanations from DEB theory (based on energetic constraints or maturation status) do not apply here, so additional mechanisms are needed. Finally, the allocation rules for the adult stage require further study and dedicated experimental work. After the final moult, gonad maturation and the gradual start up of egg production need to be explained, and the rules for fuelling of reproduction from feeding, storage and structural biomass need to be elucidated.

Using a generic framework for bioenergetics allows us to compare *C. finmarchicus* to other, even distantly related, species (see supp. info., and Jager and Ravagnan 2016). Interestingly, the life history of copepods bears striking resemblances to that of holometabolous insects, which also grow approximately exponential (on body weight basis), build up a storage over the larval stages, and stop growth after a final moult. Holometabolous insects have also recently been subject of DEB-based studies (Llandres et al. 2015; Maino and Kearney 2015), and it is likely that similar solutions to the open questions apply to both groups of animals. This underlines the power of DEB-based approaches to unify ecological research across taxa.

## Electronic supplementary material

Below is the link to the electronic supplementary material.

Supplementary material 1 (pdf 277 KB)
