# Supplementary material for: Modelling the dynamics of growth, development and lipid storage in the marine copepod Calanus finmarchicus
Source: Mar Biol. 2016 Nov 22;164(1):1. doi: 10.1007/s00227-016-3030-8 (PMC5126210; doi:10.1007/s00227-016-3030-8)
Supplement: Supplementary file 1 — Supplementary material 1 (pdf 277 KB) [file 227_2016_3030_MOESM1_ESM.pdf]

Supplements for: Modelling the dynamics of growth, development and lipid storage in the marine copepod *Calanus finmarchicus*

T. Jager*, I. Salaberria, D. Altin, T. Nordtug, B. H. Hansen

November 1, 2016

Contents

1 Conversion factors 2

2 Data removed from carbon growth curve 4

3 Ingestion rates *Calanus helgolandicus* 5

4 Comparison to *C. sinicus* 8

Bibliography 9

*DEBtox Research, De Bilt, The Netherlands, http://www.debtox.nl

1 Conversion factors

The DEBkiss model deals with body size as dry weights (mg) and surface areas (mm^2), whereas the body-size data are given as weights of nitrogen (mg N) and carbon (mg C). Furthermore, total body or prosome length (mm) is often used as a convenient measure for copepod size. To use and compare all these measures, we need to establish a consistent set of conversion factors. This task is complicated by the fact that many biometric determinations are based on field-collected animals, and only rarely the distinction between structural body and lipid storage is made. For this reason, the current set of conversion factors, summarised in Table 2, must be seen as preliminary. For the fits and predictions in this study, the absolute values of the conversion factors is not particularly important; a change in conversions will mainly change the absolute value of the parameter estimates, and not so much the model fits and predictions.

For nauplii and early copepodites, the C:N ratio is rather stable around a value of four [1]. As these stages do not have a lipid storage, we take this value as representative for structural biomass. For the fractions of carbon and nitrogen in biomass (d_C and d_N), we take 40% and 10% of the dry weight, respectively. This choice is consistent with the values reported for *C. helgolandicus* (for specimens with a C:N of 4-6, and thus with a limited contribution of lipid storage [8]), and for *Acartia tonsa* (which does not build up a lipid storage [7]).

Tande [6] reports data on wet weight and dry weight of *Calanus finmarchicus*. The dry weight density (d_V) is calculated assuming the wet density of structure to be 1 mg/mm^3). The results are plotted in Figure 1. For d_V, we take 0.27 mg mm^{-3}, which is an average of these data and corresponds well to the values for the later stages of *C. helgolandicus* [8]. The range for this parameter is very large (0.11-0.42 mg mm^{-3}, see Fig. 1), possibly reflecting the difficulties of establishing a representative wet weight for such small animals.

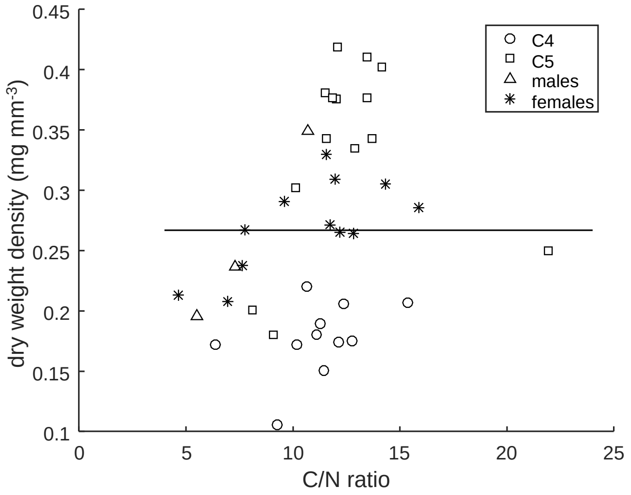

Figure 1: Dry weight density (d_V) of *C. finmarchicus* plotted against the C/N ratio. Line represents the average of all data. Data from [6].

For the yield coefficients (which translate one mass flux to another), general defaults were used [2]. The default value of 0.8 for the yield of assimilates on food (y_{AX}, the assimilation efficiency) is well within the range found for C and N in *C. pacificus* [4].

With the set of conversion factors, the dry weight of a single egg (W_{B0}) was calculated as an average from the reported C and N content in eggs [1]. From the conversion factors

Table 1: Conversion factors used in this study, which are fixed as a constant (C) or default (D) value. Note that δ_M links volumetric length to total body length of nauplii and prosome length for copepodites.

Symbol	Explanation	Value (CI) unit (status)
d_C	carbon weight per dry weight (structure)	0.40 mg mg^{-1} (C)
d_N	nitrogen weight per dry weight (structure)	0.10 mg mg^{-1} (C)
d_V	dry weight per body volume (structure)	0.27 mg mm^{-3} (C)
W_{B0}	dry weight of a single egg	0.48 μg (C)
y_{AXc}	yield of assimilates on food (carbon)	0.80 mg mg^{-1} (D)
y_{BA}	yield of egg buffer on assimilates	0.95 mg mg^{-1} (D)
y_{VA}	yield of structure on assimilates	0.80 mg mg^{-1} (D)
δ_M	shape correction coefficient (nauplii/cop.)	0.44/0.38 [−] (C)

and the reported body lengths and weights, we can also establish shape correction coefficients for nauplii and copepodites (δ_M). These coefficients can be used in the translation from volumetric lengths to physical lengths and vice versa (see model in main text).

Campbell *et al.* [1] report data for nitrogen content and phsyical length (total body length of nauplii and prosome length for copepodites). The nitrogen content can be translated to structural dry weight (using d_N), and dry weight to wet weight (using d_V), and from there to volumetric length (taking the cubic root, assuming the wet density of structure to be 1 mg/mm^3). The results are plotted in Figure 2.

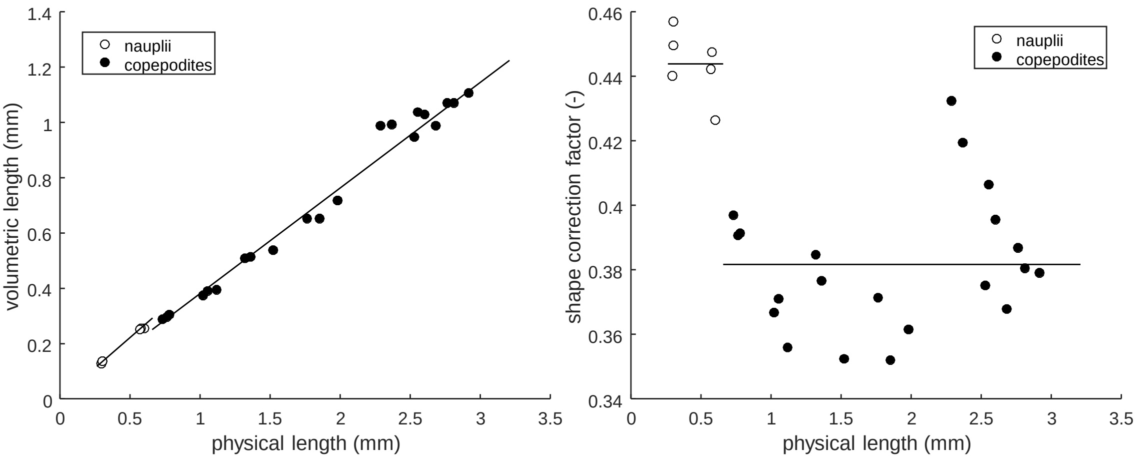

Figure 2: Left panel: volumetric length versus physical length. Lines represent the relationships for nauplii and copepodites based on the mean value of the shape correction coefficient (δ_M). Right panel: δ_M plotted versus physical length. Data from [1].

2 Data removed from carbon growth curve

As explained in the main text, several data points were excluded from the fits. These are carbon weights of adults, as the carbon content decreases over the initial part of the adult stage. There are several possible explanation for this pattern (costs for gonad maturation and use of lipid storage during reproduction), but these were not included into the model.

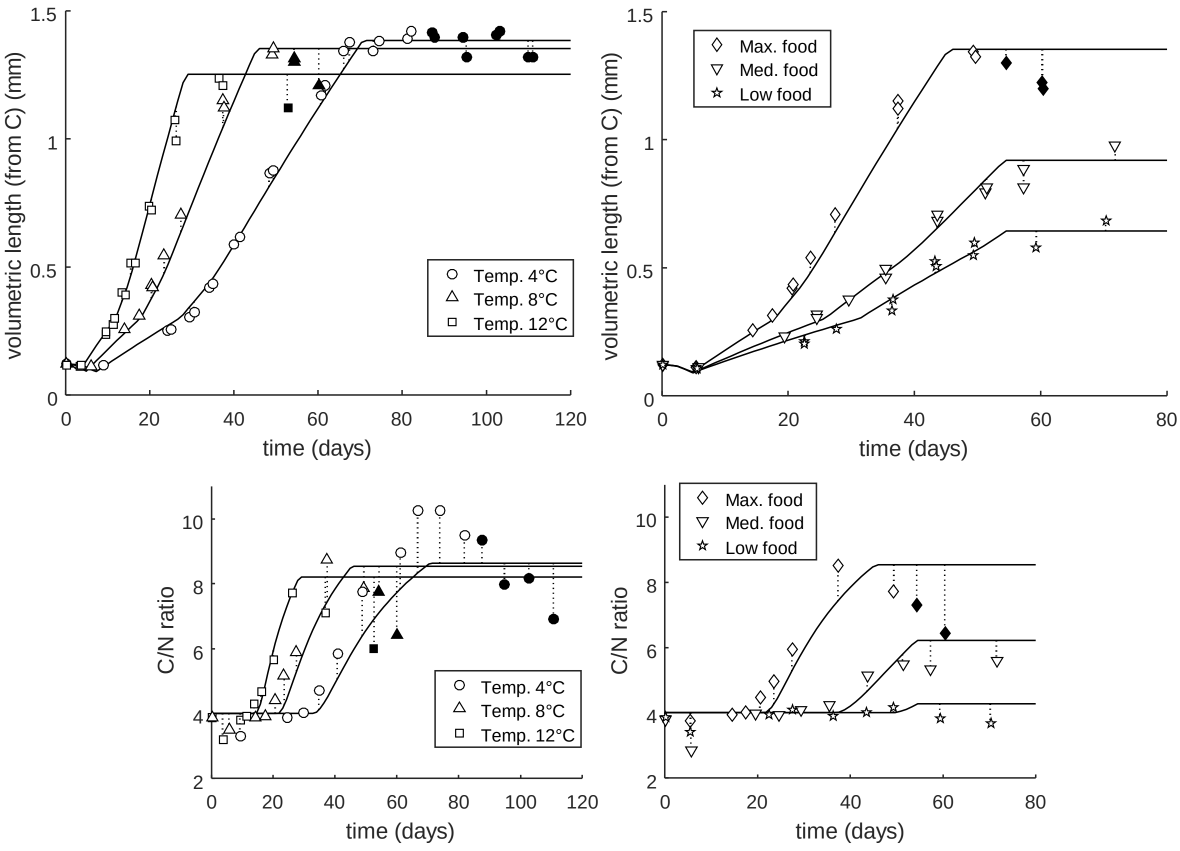

Figure 3: Top figures: carbon content versus age at different temperatures (left) and food levels (right). Lower figures: C/N ratio versus age at different temperatures (left) and food levels (right). Filled symbols have been excluded from the fits. Data from [1].

3 Ingestion rates *Calanus helgolandicus*

Paffenhöfer [5] presents detailed ingestion rates for a different calanoid species, *Calanus helgolandicus*. Ingestion rates in the original publication were expressed in μg carbon per day, plotted versus total body weight in μg carbon. The first problem with these data is that they are for a different, though related, species. Secondly, the carbon body weights have been estimated from the dry weight using stage-specific conversion factors from a different study. And thirdly, the total body weight will include an unknown contribution from the lipid sac.

In Figure 4, the ingestion rates are plotted versus the estimated volumetric length (using the conversion factors from Table 2). The data are the observations for *C. helgolandicus* and the thick line is the model estimate for *C. finmarchicus*.

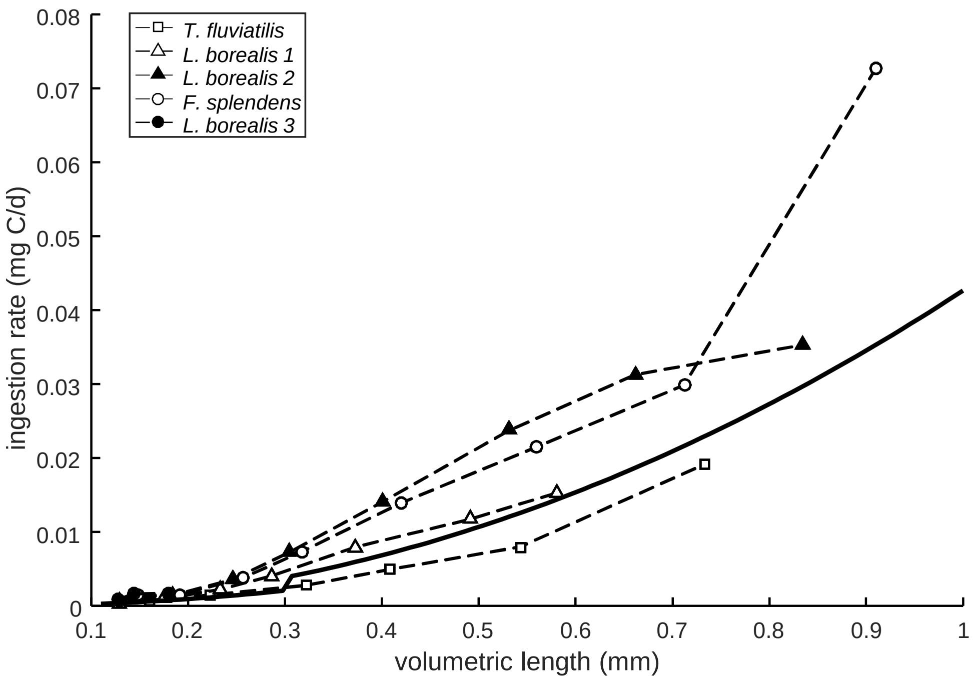

Figure 4: Ingestion rate data from [5] versus estimated volumetric length. Different symbols represent different food types (same symbols as in original publication). Thick line represents the model estimate for *C. finmarchicus*.

As this plot is difficult to interpret, we first focus on two food types producing the highest and lowest feeding rates (Fig. 5). An additional model line is shows (the estimate times a factor of two) to provide an approximate fit to the treatment with higher feeding rates. The model pattern is reasonably consistent with the data, although the low treatment shows less acceleration than predicted. The high treatment shows a decreasing trend in the ingestion rates at larger body sizes, which likely relates to the build up of the lipid sac (the amount of carbon in structure is less than the total amount of carbon, so these data points can be shifted to the left).

Next, we plot two treatments with high observed feeding rates, and further zoom in on the switch as used in the model (Fig. 6). Although the data do indicate an acceleration of the ingestion rates, it appears that it could be a more gradual process over the first few copepodite stages.

It is difficult to draw firm conclusions based on these data. In general, they support

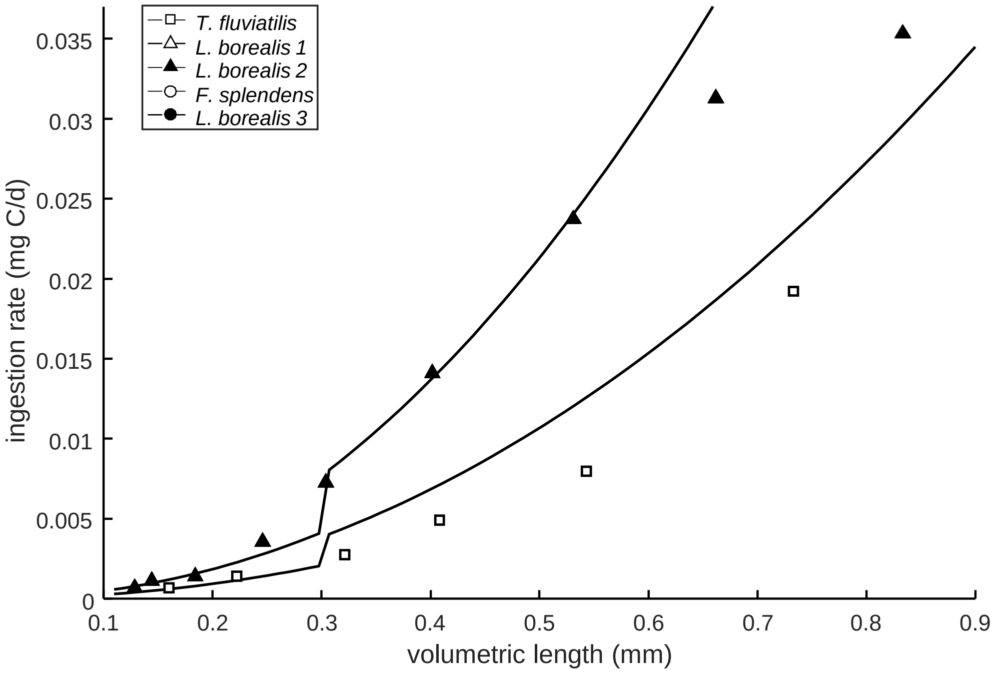

Figure 5: Ingestion rate data from [5] versus estimated volumetric length. Different symbols represent different food types (same symbols as in original publication). Lines represent model estimates for *C. finmarchicus*; top line is the estimate times a factor of two.

an acceleration of the ingestion rate over ontogeny. However, they indicate that a more gradual increase over the first few copepodite stages might be more realistic. Furthermore, this data set indicates that these patterns may also depend on the food source that is provided.

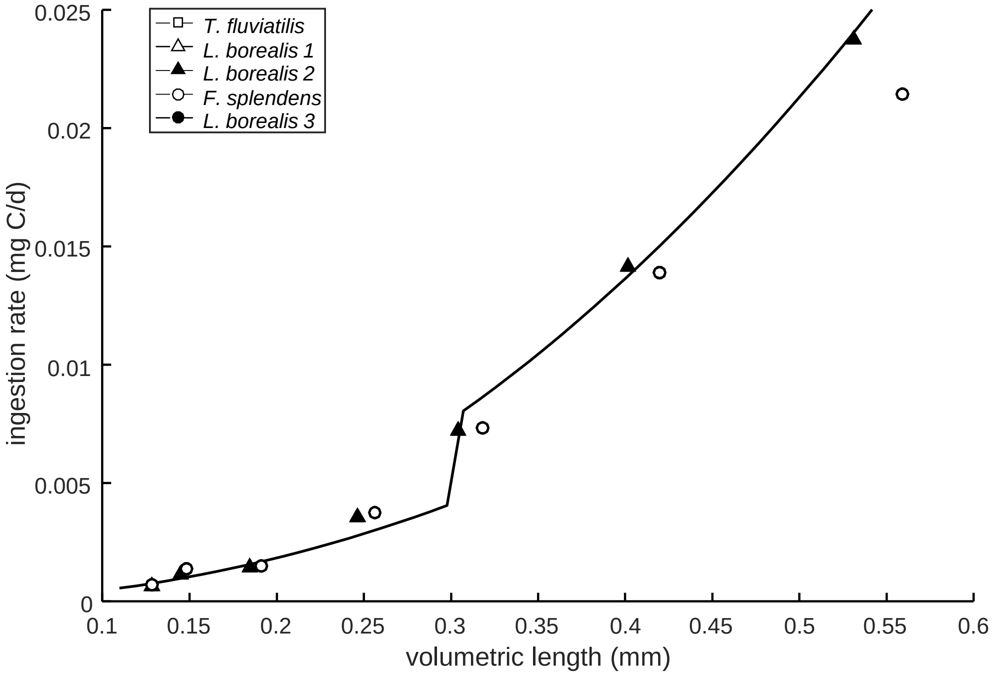

Figure 6: Ingestion rate data from [5] versus estimated volumetric length. Different symbols represent different food types (same symbols as in original publication). Line represent model estimates for *C. finmarchicus* times a factor of two.

4 Comparison to *C. sinicus*

C. sinicus was fitted with DEBkiss previously [3]. For both species, the reference temperature was set to 10°C, so we can directly compare the model parameters. *C. finmarchicus* is somewhat larger than *C. sinicus* (roughly 1 versus 0.8 mm volumetric length), but otherwise very similar. The somatic maintenance rate coefficient (J^v_M) is a factor of two lower for *C. finmarchicus*. In the current analysis, we used respiration data to constrain this parameter's value, but in the study for *C. sinicus* it was based on the (very limited) curvature of the growth data. The available respiration data for *C. sinicus* were overestimated by roughly a factor of two, so we should consider that the lower value established in this study may be more realistic for both species.

The specific assimilation rate (J^a_{Am}) is more than a factor of two higher for *C. finmarchicus*. This likely relates to the low value of κ established in this study (close to 0.5) versus the default value of 0.8 used for *C. sinicus*. A low value of κ implies that a larger fraction of the assimilated energy is allocated towards maturation and lipid storage. To allow for a rather similar growth curve, a higher assimilation rate would be needed. For *C. finmarchicus*, we need a low κ because of the very high value for the maturity maintenance costs (J^v_J), which in turn was needed to catch the limited lipid build up at low food levels. Clearly, all parameters are tightly linked to each other and to the choice of model structure. Because several metabolic aspects of the copepod life history remain to be elucidated, discussing and comparing parameter values is still rather speculative.

Table 2: Comparison of conversion factors and model parameters between the two copepod species (n.a. is not applicable), which are fixed as a constant (C) or default (D) value, or fitted (F). Confidence intervals for fitted parameters are approximate 95% intervals by profiling the likelihood function. All rate constants are referenced to 10°C. Maximum length (L_a) for *C. finmarchicus* is at 8°C and maximum food. For *C. sinicus*, egg weight (W_{B0}) was estimated from the body size data post hatch.

Symbol	*C. sinicus*	*C. finmarchicus*	unit
d_C	0.45 (C)	0.40 (C)	mg mg^{-1}
d_N	n.a.	0.10 (C)	mg mg^{-1}
d_V	0.26 (C)	0.27 (C)	mg mm^{-3}
J^a_{Am}	0.036 (0.034-0.038) (F)	0.0852 (0.0832-0.0870) (F)	mg mm^{-2} d^{-1}
J^v_J	0 (n.a.)	0.268 (0.206-0.281) (F)	mg mm^{-3} d^{-1}
J^v_M	0.020 (0.018-0.024) (F)	0.0106 (0.00982-0.0113) (F)	mg mm^{-3} d^{-1}
L_0	0.00073 (D)	0.01 (D)	mm
L_p	n.a.	0.303 (0.295-0.308) (F)	mm
L_a	0.78 (0.76-0.79) (F)	1.05 (1.02-1.08) (F)	mm
T_A	7700 (7400-8000) (F)	8200 (8020-8380) (F)	K
W_{B0}	0.72 (0.62-0.86) (F)	0.48 (C)	μg
y_{AXc}	0.80 (D)	0.80 (D)	mg mg^{-1}
y_{BA}	0.95 (D)	0.95/0.4 (D)	mg mg^{-1}
y_{VA}	0.80 (D)	0.80 (D)	mg mg^{-1}
δ	1 (n.a.)	0.535 (0.522-0.547) (F)	[-]
δ_M	0.50/0.37 (C)	0.44/0.38 (C)	[-]
κ	0.80 (D)	0.483 (0.464-0.493) (F)	[-]

References

[1] R. G. Campbell, M. M. Wagner, G. J. Teegarden, C. A. Boudreau, and E. G. Durbin. Growth and development rates of the copepod *Calanus finmarchicus* reared in the laboratory. *Marine Ecology Progress Series*, 221:161–183, 2001.

[2] T. Jager, B. T. Martin, and E. I. Zimmer. DEBkiss or the quest for the simplest generic model of animal life history. *Journal of Theoretical Biology*, 328:9–18, 2013.

[3] T. Jager, I. Salaberria, and B. H. Hansen. Capturing the life history of the marine copepod *Calanus sinicus* into a generic bioenergetics framework. *Ecological Modelling*, 299:114–120, 2015.

[4] M. R. Landry, R. P. Hassett, V. Fagerness, J. Downs, and C. J. Lorenzen. Effect of food acclimation on assimilation efficiency of *Calanus pacificus*. *Limnology and Oceanography*, 29(2):361–364, 1984.

[5] G. A. Paffenhöfer. Grazing and ingestion rates of nauplii, copepodids and adults of marine planktonic copepod *Calanus helgolandicus*. *Marine Biology*, 11(3):286–298, 1971.

[6] K. S. Tande. Ecological investigations on the zooplankton community of Balsfjorden, northern Norway: generation cycles, and variations in body weight and body content of carbon and nitrogen related to overwintering and reproduction in the copepod *Calanus finmarchicus* (Gunnerus). *Journal of Experimental Marine Biology and Ecology*, 62(2):129–142, 1982.

[7] J. Walve and U. Larsson. Carbon, nitrogen and phosphorus stoichiometry of crustacean zooplankton in the Baltic Sea: implications for nutrient recycling. *Journal of Plankton Research*, 21(12):2309–2321, 1999.

[8] R. Williams and D. B. Robins. Effects of preservation on wet weight, dry weight, nitrogen and carbon contents of *Calanus helgolandicus* (Crustacea, Copepoda). *Marine Biology*, 71(3):271–281, 1982.
